# 急性淋巴细胞白血病微小残留病检测与临床解读中国专家共识（2023年版）

**DOI:** 10.3760/cma.j.issn.0253-2727.2023.04.002

**Published:** 2023-04

**Authors:** 中华医学会血液学分会实验诊断学组

急性淋巴细胞白血病（ALL）是一种淋系细胞分化被阻滞在干/祖细胞阶段的血液系统恶性疾病，以白血病细胞侵犯骨髓、外周血和髓外组织为特征[1]–[2]。近年来，随着儿童样化疗方案在成人中的应用、小分子靶向药物和免疫治疗新方法的应用以及异基因造血干细胞移植（allo-HSCT）技术的进步，ALL患者的临床预后得到极大改善[3]–[10]。然而，复发仍是限制ALL疗效提高的主要因素[4],[11]。研究表明微小残留病（MRD）不仅能用于评估ALL患者的疗效、预测复发，还能指导治疗方案的选择[11]–[14]；关于MRD的概念请参见《急性髓系白血病微小残留病检测与临床解读中国专家共识（2021年版）》[15]。目前，我国在ALL患者MRD检测领域仍存在不同诊疗中心应用的检测方法各异、标准化程度待提升以及检测结果解读欠标准等问题。为规范MRD在ALL诊疗中的应用，进一步提高ALL患者的诊治水平，中华医学会血液学分会实验诊断学组召集国内ALL领域从事实验诊断和临床诊疗工作的相关专家，制定了ALL患者MRD检测与临床解读的中国专家共识，具体如下。

一、多参数流式细胞术（MFC）检测ALL患者MRD的临床解读

与急性髓系白血病（AML）相似[15]，利用MFC识别ALL患者MRD的方法包括白血病相关异常表型（LAIP）以及与正常骨髓细胞表型相鉴别（different from normal，D-F-N）（其敏感性及优势见表1和图1）。与AML不同的是，目前尚缺乏基于ALL的白血病干细胞检测MRD的相应标志。专家组推荐：联合应用LAIP与D-F-N两种方法检测已知和（或）验证未知的、具有预后意义的白血病细胞表型异常。

**表1:** 急性淋巴细胞白血病（ALL）各种MRD检测方法的比较

检测方法	标本	敏感性	优势	劣势
多参数流式细胞术（LAIP或D-F-N）	新鲜的细胞标本	10^−4^	快速 相对便宜 可发现表型漂移 可不需要治疗前标本 覆盖95%以上的患者	骨髓恢复时良性B前体细胞的干扰 需要经验丰富的流式细胞术专家 缺乏标准化 使用4~6色流式细胞术时敏感性受限
RQ-PCR检测Ig/TCR基因重排	DNA	10^−4^~10^−5^	敏感 可实现不同实验室之间的标准化 覆盖90%以上的患者	耗时耗力 需要经验丰富的分子技术专家 初诊时可能不能发现小克隆 需要治疗前标本 昂贵
RQ-PCR检测重现性融合基因	RNA	10^−4^~10^−5^	敏感 使用诊断目的标准化引物	只可应用于35%~45%的ALL 标准化受限
NGS检测Ig/TCR基因重排	DNA	10^−6^	高度敏感 快速（使用通用型引物） 覆盖90%以上的患者；该方法可追踪小的白血病亚克隆、发现白血病细胞克隆演变	未标准化 需要复杂的生信分析 临床可及性不够 需要治疗前标本 昂贵

**注** MRD：微小残留病；LAIP：白血病相关免疫表型；D-F-N：与正常骨髓细胞表型相鉴别；RQ-PCR：实时定量聚合酶链反应；NGS：二代测序

**图1:**
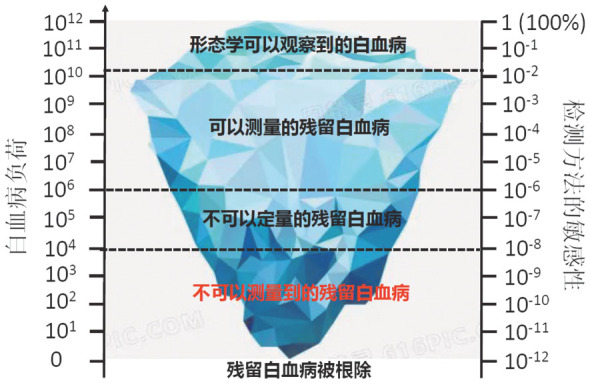
急性淋巴细胞白血病微小残留病评估

（一）检测技术要求

1. 流式细胞仪：专家组推荐应用至少八色荧光标记的MFC对标本进行MRD检测，以提高敏感性和特异性。不同实验室或同一实验室不同仪器应遵循标准化操作流程，具体参见相关文献[15]–[16]。

2. 抗体组合和荧光标志：专家组建议应用完整的抗体组合方案评估ALL患者的MRD[13],[16]–[18]：

（1）B-ALL：①骨架抗体有CD10、CD19、CD20、CD34、CD38和CD45；②其他抗体：CD13、CD15、CD33、CD58、CD65、CD66c、CD73、CD81、CD86、CD123、CD304以及NG2等。专家组推荐的八色MFC抗体组合如下：CD58-FITC、CD38-PE-Cy5.5、CD34-PE-Cy7、CD10-APC、CD20-APC-H7（或APC-Cy7或APC-AlexaFlour750）、CD19-Bv421、CD45-Pacific Orange，PE通道可选择前述其他抗体中的任何一种；对于配备十色MFC的中心，可针对PE、Bv605/ECD或APC-R700三个检测通道从可选择抗体中调配MRD检测十色抗体组合。

（2）T-ALL：①骨架抗体有mCD3、cyCD3、CD7和CD45以及nTdT或CD99；②其他抗体：CD2、CD4、CD5、CD8、CD1a、CD10、CD34、CD13、CD33、CD117、CD11b、CD65以及nTdT等。专家组推荐的八色MFC抗体组合如下：CD99-PE、CD3-PE-Cy5.5（或Per-CP-Cy5.5）、CD7-PE-Cy7、CD5-APC-H7（或APC-Cy7或APC-AlexaFlour750）、cyCD3-Bv421、CD45-Pacific Orange，FITC和APC通道可选择前述其他抗体中的任何一种；对于配备十色MFC的中心，可针对FITC、APC、Bv605/ECD或APC-R700三个检测通道从可选择抗体中调配MRD检测十色抗体组合。

（3）接受CD19 CAR-T细胞治疗的B-ALL：①骨架抗体有CD10、CD20、CD34、CD79a和CD45[19]–[22]；②其他抗体：CD13、CD19、CD22、CD33、CD81、CD86、CD123、CD66b以及CD58等。美国华盛顿大学Wood教授实验室应用八色组合检测针对CD19靶点治疗后的B-ALL MRD抗体组合为CD66b-FITC、CD22-PE、CD34-PerCP-CY5.5、CD20-PE-CY7、CD38-A594、CD24-APC、CD45-APC-H7、CD10-BV421[20]。对于接受CAR-T细胞治疗的患者，同时采用MFC和分子学方法检测MRD可部分避免假阳性或假阴性。

对于应用FCM检测MRD的患者，应该通知实验室患者是否接受了针对CD7、CD19、CD20、CD22和CD38等靶抗原的CAR-T细胞或单克隆抗体或双特异性抗体等治疗；该类MRD检测一定要在有经验的实验室进行。

3. 标本采集储存、样本抗体标记与检测：参见《多参数流式细胞术检测急性白血病及浆细胞肿瘤微小残留病中国专家共识（2017年版）》[16]。

（二）MRD的识别和评估

MFC检测的ALL患者MRD识别和评估参见《多参数流式细胞术检测急性白血病及浆细胞肿瘤微小残留病中国专家共识（2017年版）》[16]。

MRD检测敏感性的主要决定因素包括：在应用二代流式细胞术的条件下，欧洲流式细胞术工作组（EWG）推荐[17]，对于B-ALL而言，如果获取的细胞数量超过400万个有核细胞，那么MFC检测MRD的敏感性可达10^−5^。EWG定义MRD阳性为10个集群的具有异常表型的B祖细胞（LOD），40个细胞被该工作组推荐为可重复定量检测的最低细胞数（LOQQ）。我国学者推荐：MRD阳性被定义为20个集群的具有异常表型的B祖细胞（LOD），50个细胞被推荐为可重复定量检测的最低细胞数（LOQQ）[16]。

（三）MFC检测的MRD报告方式

出具MFC检测的MRD报告时应注意事项参见《多参数流式细胞术检测急性白血病及浆细胞肿瘤微小残留病中国专家共识（2017年版）》[16]。专家组建议：在出具的检测报告中必须清晰描述获取细胞数量及MFC检测到的MRD水平，即残留白血病细胞占骨髓或外周血有核细胞的比例。

（四）MFC检测的MRD临床解读

1. MRD阈值的确定：目前多数文献将MFC检测ALL患者MRD的预测复发阈值定为0.01％[13],[17],[23]–[25]。由于预测白血病复发的阈值受到治疗方案、检测时间点和标本类型等多种因素的影响，国内外学者关于ALL患者MRD的最佳阈值仍存在争议[26]–[28]。专家组建议：应积极开展前瞻性、多中心、大样本临床研究以确定不同场景下MFC检测到的、预测ALL复发的MRD最佳阈值。

2. 预后预测和指导治疗：①诱导治疗第15天、诱导或巩固治疗后以及HSCT前后，MFC检测MRD阳性均提示高复发率、预后不良[13],[26],[29]–[30]。②诱导或巩固治疗后MFC检测MRD阳性的患者可选择allo-HSCT以改善预后[31]。③我国学者制定的造血干细胞移植专家共识推荐[4]：对于移植前MFC检测MRD阳性的ALL，可以选择贝林妥欧单抗治疗后再行allo-HSCT（有经验的移植中心可以首选单倍体HSCT）。④移植后MRD指导的抢先干预（例如干扰素α-2b、供者淋巴细胞输注、贝林妥欧单抗等免疫或细胞治疗）可以降低血液学复发率，改善移植预后[32]。

诊断学组专家还推荐：①经验缺乏的中心不宜开展MFC检测MRD项目；②逐步通过多中心合作实现MFC检测MRD的标准化、规范化；③利用人工智能技术实现MFC检测的MRD结果分析的自动化很重要。

二、分子生物学方法检测ALL患者MRD的临床解读

（一）分子生物学检测技术

目前，检测ALL的MRD分子生物学方法主要包括：实时定量聚合酶链反应（RQ-PCR）、二代测序技术（NGS）以及数字PCR（ddPCR）技术等，其敏感性及优势见表1和图1。

1. RQ-PCR：可采用RQ-PCR检测B细胞受体（BCR）重排/T细胞受体（TCR）基因克隆性重排和重现性白血病相关的融合基因。

（1）Ig/TCR基因重排：Ig/TCR是正常B细胞和T细胞早期成熟过程中的随机生理事件，而克隆性的Ig和TCR重排是恶性淋巴细胞的标志。PCR直接检测的是多样性V、（D）、J区的重排，在欧洲应用较为普遍，相对于北美常用的MFC方法检测MRD，敏感性约可提升1个log[33]–[34]。对于B-ALL和T-ALL，RQ-PCR方法可用于检测每个患者特有的Ig/TCR重排连接区，初诊时所设计的患者特异性探针用于治疗后标本的检测，评估MRD水平，这种方法可以用于90％～95％的ALL患者[35]–[36]。该种方法尽管在Euro-MRD协作组已经标准化，但在美国仍然没有实现标准化，此外该种方法较难用于早前T-ALL患者MRD检测，因为这种类型白血病细胞以不成熟淋巴细胞为主，大多尚未经历TCR重排，可能出现假阴性结果[37]。

（2）重现性ALL特异融合基因：

①Ph^+^ ALL：对于Ph^+^ ALL，BCR-ABL融合基因转录本是可靠的MRD监测指标[38]–[39]；使用RQ-PCR可监测BCR-ABL转录本水平，该方法快速、简单、敏感。尽管BCR-ABL的P210转录本的定量检测作为评估慢性髓性白血病（CML）疗效可靠的分子指标，已经得到了较好的标准化，但其对CML的监测意义并不能扩展到ALL的疗效评估中[40]。极少数患者的BCR-ABL转录本可在非ALL的造血细胞中被发现[41]，而研究证明这种表现为“CML样”的Ph^+^ ALL，残留的BCR-ABL表达并不一定影响预后[42]。

②Ph^−^ALL：除了BCR-ABL，儿童ALL中最为常见的融合基因为ETV6-RUNX1，占儿童ALL的25％～30％。其他融合基因如KMT2A-AFF1和TCF3-PBX1各占ALL的3％～8％。婴儿（小于1岁）的KMT2A重排占婴儿ALL的80％。此外B-ALL相关融合基因还包括IGH-IL3、TCF3-HLF等。T-ALL中，TAL1缺失（SIL-TAL1）发生率为20％左右。由于这些染色体易位通常具有临床预后意义，因此，在所有ALL初始诊断时建议筛查上述融合基因，阳性患者所涉及的融合基因可用于后期MRD的监测随访。

随着RNA测序技术在临床中的应用，在ALL中可以发现更多的已知/未知的融合基因，包括Ph样ALL（Ph-like）相关的多种融合基因，理论上这些融合基因均可用于MRD的监测，但每种融合基因根据其探针引物设计的不同，初诊表达水平的差异，以及其表达水平随肿瘤负荷变化的动力学特征仍不明确，还需要积累临床数据确认，以便后期形成标准化试剂盒进行临床MRD的监测。

2. NGS：在ALL患者中，基于NGS技术所监测的目标为与初诊一致的白血病克隆特异性的IGH和TCR基因重排[43]–[45]。NGS使用一致的引物进行平行、快速测序，它不需要订制患者特异性引物、探针，因此这种技术更容易实现标准化。一些临床研究证实NGS检测MRD与MFC或PCR技术有着很高的一致性。理论上NGS技术监测MRD敏感性可以高出其他MRD监测方法（RQ-PCR或MFC）1～2个log[46]–[47]。NGS相比于PCR方法检测ALL的MRD更为特异。

此外，NGS还可用于追踪其他方法不易发现的微小亚克隆，这些小克隆可能是后期ALL复发的主要原因之一[48]。尽管理论上NGS检测MRD有以上很多优势，但达到10^−6^的敏感性需要患者缓解时足够量的骨髓血/DNA，限制了它在很多临床场景的应用。对于治疗后骨髓增生不良的标本，NGS有可能过度扩增了非恶性的重排而高估了MRD水平。2018年9月28日美国食品药品管理局（FDA）批准cloneSEQ NGS试剂盒（美国Adaptive Biotechnologies公司）可用于ALL和多发性骨髓瘤MRD的检测，这是第一个获批的用于淋系血液恶性肿瘤MRD检测的NGS试剂盒。

3. ddPCR：ddPCR技术可对样本中特定DNA分子进行绝对定量，而无需标准曲线的制作。ddPCR降低了非特异性靶标竞争和抑制剂的影响，因此不需要对目标基因建立标准曲线。利用该项技术对IgH/TCR基因重排或BCR-ABL转录本进行定量检测，国外研究结果提示：ddPCR与常规RQ-PCR具有高度一致性，且可以对RQ-PCR的阳性但不可定量（positive non-quantifiable，PNQ）范围的MRD进行更准确的预后分层。同时由于ddPCR理论上比RQ-PCR检测敏感性高1个log，也成为潜在的使用外周血监测ALL-MRD的方法之一[49]–[51]。

专家组推荐：①对于伴有融合基因的ALL，优先使用融合基因进行MRD监测；②对于不伴融合基因的ALL，可以使用NGS识别Ig/TCR的MRD靶标，随后针对靶标设计特异性探针通过RQ-PCR方法进行MRD监测；③无论有无融合基因，均可通过NGS方法进行Ig/TCR MRD监测，此方法优于Ig/TCR RQ-PCR法。鼓励国内学者开展多中心、前瞻性研究评估NGS或ddPCR用于MRD检测较现有技术的优缺点。

（二）分子标志物检测MRD的技术要求

专家组推荐cDNA而非DNA用于基因检测；BCR-ABL、TCF3-PBX1、KMT2A-AFF1和SIL-TAL1融合基因的检测具体方法参见相关文献[52]–[53]。对于Ig/TCR的MRD监测，需要使用DNA。根据欧洲抗癌计划标准[52]–[53]，每个样本应同时进行3个反应，PCR结果定义为阳性需要3个重复反应中至少2个反应Ct值≤40［循环阈值（CT）为0.1］。作为对照，专家组建议同时包括野生型样品（正常对照），以及至少2个覆盖所需灵敏度范围的阳性对照和非目标对照（水对照）。如果阳性对照是从质粒产生的，则定期监测质粒稳定性。

PCR方法检测MRD时，推荐应用1 µg RNA逆转录为cDNA，每次反应的cDNA相当于100 ng RNA（约为200 000个细胞，如果基因表达水平高则需要的细胞数量少）。对于基于DNA的方法，每次反应需要至少100 ng DNA（约为15 000个细胞），理想目标是达到每次反应1 000 ng（约为150 000个细胞）。管家基因ABL的拷贝数至少应该为10 000拷贝（无论是基于RNA还是DNA的方法）。不过，1 000～9 999 ABL拷贝数的管家基因数量也可以报告MRD结果，但需要特别标明管家基因拷贝数低于理想值[52]–[53]。

MRD从阴性转为阳性后，应使用2种方法来控制重复样品中的检测变异性：其一，在复核检测的样本中，应包括可疑分子复发的初始样本；其二，如果MRD检测采用RQ-PCR方法，标准曲线应覆盖到患者样本可能的CT值范围，以确保检测的MRD水平在此线性范围内。如果MRD结果为阴性，确认检测的敏感性至关重要[52]–[53]。在计算单独一次RQ-PCR结果的敏感性时建议应用下面的公式，它可用于绝对定量，即利用外源性质粒标准品估计目标分子的个数，和相对定量一样。

X＝［（CT_目标基因_−CT_ABL_）_FU_−（CT_目标基因_−CT_ABL_）_诊断_］/斜率

方法的敏感性=10^X^

ABL＝管家基因ABL；诊断=诊断时进行MRD检测；FU＝随访时进行MRD检测；斜率=标准曲线的斜率，如果方法的效率为100％，则斜率为−3.32；目标基因＝MRD检测的目标基因。

（三）分子学MRD结果报告方式

各临床中心血液诊断实验室出具分子学MRD报告时应注意如下事项：①写明MRD检测的靶基因，如BCR-ABL、TCF3-PBX1、KMT2A-AFF1等；②标明所应用的技术，如RQ-PCR；③标明标本来源，如骨髓或外周血等；④标本质量是否适合行MRD检测；⑤目的基因拷贝数，如BCR-ABL基因的拷贝数和CT值；⑥内参基因拷贝数，如ABL基因；⑦目的基因相对于内参基因百分比，如BCR-ABL拷贝数/ABL拷贝数×100％；⑧标明检测方法的敏感性等。此外，有条件的单位可以给出所检测基因预测ALL患者治疗后复发的阈值，并就下次复查的时间给出建议。

（四）分子学MRD的临床解读

1. 分子学MRD的概念：

（1）完全分子学缓解（Complete molecular remission, CR_MRD_^−^）：定义CR_MRD_^−^的前提是患者必须获得血液学完全缓解（HCR），连续两次分子学MRD阴性，标本采集间隔时间≥4周，检测方法敏感性至少为10^−3^。

（2）低水平分子标志持续存在：是指患者处于HCR，分子生物学MRD标志持续低水平存在（小于100～200拷贝/10^4^ ABL拷贝，相当于靶基因与参考基因比值小于1％～2％）和治疗结束后任何两次阳性标本之间基因检测的拷贝数相对上升小于1个log。

（3）分子学进展：低水平分子标志持续存在患者，任何两份阳性标本之间MRD标志基因检测拷贝数升高≥1个log。

（4）分子学复发：患者处于HCR且CR_MRD_^−^后，再次出现MRD阳性，两份阳性标本之间MRD水平上升≥1个log。

2. 临床解读：

（1）Ph^+^ALL的MRD：在评估BCR-ABL作为MRD的意义前，首先要明确区分Ph^+^ALL和CML急变期，因BCR-ABL转录本水平作为Ph^+^ALL的MRD更为恰当。从诱导化疗开始，获得CR_MRD_^−^的时间早晚对患者的预后至关重要。如果在治疗后3个月获得CR_MRD_^−^，可选择继续化疗联合酪氨酸激酶抑制剂（TKI）治疗，也可选择allo-HSCT，并在移植后用TKI维持治疗。如患者在治疗后3个月未能达到CR_MRD_^−^，则需选择allo-HSCT。在移植前BCR-ABL转录本越低，移植后复发率越低。移植前可考虑给予2～4个疗程CD3/CD19双抗联合/不联合TKI，以期在移植前达到CR_MRD_^−^。BCR-ABL转录本水平低于0.1％被认为与移植后较低的复发率相关。同样，移植后早期BCR-ABL转录本水平<0.01％（移植后3个月内）与低复发率相关[34]。

（2）Ph^−^ALL的MRD：目前，MFC仍然是我国主要并公认的Ph^−^ALL患者的MRD监测方法。分子学检测主要基于伴有重现性的遗传学异常，如采用RQ-PCR方法检测ALL相关融合基因，包括TCF3-PBX1、ETV6-RUNX1、KMT2A重排以及Ph样ALL相关等过表达或融合基因。但由于各单位所采用试剂盒不尽相同，标准化程度仍然不够，各个融合基因表达水平的动力学变化与疾病进程的相关性也有所不同，因此在临床中可用于MRD监测，但基于这些检测进行复发干预的阈值仍需进一步探讨。

三、MRD评估的时间点和标本选择

（一）ALL患者MRD评估的时间点

1. 初诊ALL患者：在初始治疗前，应留取骨髓标本作为MRD评估的基线值，诱导治疗第15天、诱导缓解后、巩固治疗后（约治疗后的3个月内），以及后期每间隔3个月应评估MRD，直至3年。对于Ph^+^ ALL患者如未在第1次缓解期接受移植，应至少监测MRD 3～5年。对准备接受allo-HSCT的患者，在移植前1个月内应再次评估MRD，移植后推荐在+1、+2、+3、+4.5、+6、+9个月及+12个月的时间点监测骨髓MRD水平，移植1年后每3个月评估骨髓MRD情况直至移植后3年，期间根据病情变化酌情评估骨髓MRD。

2. 复发/难治ALL患者：对于接受挽救治疗的复发/难治ALL患者，MRD应在形态学缓解后及治疗结束时进行评估，特别是对首次接受挽救治疗的患者意义重大。

此外，无论在接受何种治疗后，临床医师考虑患者疾病有变化时，可随即对患者进行MRD评估。

（二）ALL患者MRD标本选择

在B-ALL中，外周血中MRD水平往往低于骨髓1～3个log，因此仍然建议使用骨髓来评估MRD；而在T-ALL中，无论儿童还是成人，外周血是MRD监测标本的可靠来源，与骨髓的MRD结果无显著差异[54]。尽管如此，鉴于缺乏大样本、前瞻性、多中心研究，专家组推荐：使用骨髓评估B-ALL和T-ALL患者的MRD。

四、MRD监测方法的选择

对于不同的ALL类型，可选择不同的MRD监测方式。Ph^+^ALL患者采用RQ-PCR方法检测BCR-ABL转录本水平优于MFC。对于Ph^−^的B-ALL和T-ALL，根据各实验室所能具备的检测平台和技术选择使用MFC、RQ-PCR或NGS检测MRD。而early T-cell precursor ALL（ETP-ALL）由于发生在不成熟的前体细胞，Ig/TCR的重排尚未开始，因此绝大多数ETP-ALL并不能用Ig /TCR来监测MRD。

结合中国国情，专家组推荐[33]–[34],[55]：①MFC和RQ-PCR仍然是主要的ALL-MRD监测手段，两种方法应联合应用；②NGS或ddPCR技术检测ALL患者MRD仍在临床试验阶段，还需要进一步标准化才能得以推广应用。

五、ALL患者MRD检测的其他推荐

（一）临床试验中的MRD检测

无论是化疗、靶向治疗、免疫治疗还是allo-HSCT场景下，获得HCR后MRD阳性的ALL患者都是复发高危人群；专家组建议对纳入临床试验的患者，应遵循该共识的推荐方法在疗效评估的每个时间点进行MRD检测[56]–[59]。MRD在ALL患者临床治疗过程中的预测复发、指导治疗方案选择的价值已经很明确，但是在ALL临床试验中将MRD作为替代研究终点还需更多的证据[60]；专家组建议在获得足够的循证依据之前，不应将MRD作为ALL患者临床试验的替代终点。

（二）据MRD选择药物适应证

2018年3月29日，FDA批准贝林妥欧单抗（blinatumomab）可用于获得形态学缓解，而MRD仍阳性的B-ALL患者的治疗[61]。

六、小结

鉴于MRD在ALL患者疗效评估、复发预警以及指导干预和治疗方法选择等方面的价值，MRD检测已经被写入欧美、中国等专家制定的各种ALL诊治共识[4],[10],[62]–[63]。无论是在欧美国家，还是我国，在规范MRD检测、准确解读MRD的临床意义并指导个体化治疗方面仍有许多问题亟待解决。可以预见，随着今后在ALL诊疗领域MRD基础、转化以及临床研究和实践的不断进展，本共识将不断更新、逐步完善。
